# Observations on Sulphuric Æther and Its Preparation

**Published:** 1809-11

**Authors:** 

**Affiliations:** Pharmaceutist of Paris


Some of our Correspondents having expressed a wish for a Description of the new Process of procuring the Sulphuric Æther, we have translated the Article at length from the *Annales de Chimie.*

---

*Observations on Sulphuric Æther and its Preparation.* By Mons. BOULAY, Pharmaceutist of Paris. Read at the Society of Pharmacy, May 15, 1807.

THE use of sulphuric æther is now so extensive, and its consumption so considerable, that it affords a fair scope for the manufacturer in the large way. But its manipulation, though highly simple, is well worthy attention, and appears capable of amelioration, both with respect to œconomy and the purity of the product.

In its formation, whether by simple distillation of a mixture of concentrated sulphuric acid and alcohol, or by the addition of fresh alcohol to the residuum, the whole of the product is not equally sweet, and in spite of careful rectifications, the last portions always retain an odour more or less disagreeable, and which is to be attributed to the oil in intimate union, from which it is almost impossible to separate it completely.

From the learned researches, and the theory given by Messrs. Fourcroy and Vauquelin on this fluid, it appears, that the attraction of sulphuric acid for water, aided by heat, causes the transformation of alcohol into æther. This

This re-action of the principles of alcohol, exercised under the influence of the sulphuric acid, precedes the carbonization of the mixture, the formation of the sweet oil (oil of wine), the disengagement of the sulphurous acid, and the other phenomena which arise on pushing the operation to the end. We may rest assured that no farther formation of æther will take place when these products make their appearance; and that whatever passes afterwards is nothing more than what is separated from the residue which contained it in its formation. It will be advisible then to oppose, or at least to protract the appearance of these products, which announces the complete decomposition of the alcohol; and by the addition at proper times, of fresh quantities of this liquid, to keep up the proportions, so that the ætherization may continue to a longer period. In order to produce this effect, it appears necessary that the sulphuric acid should never exceed two-thirds of the quantity contained in the retort, and that the proportion of alcohol should never be less than that of the remaining third.* By this means we prevent the sulphuric acid from burning the alcohol and its constituents, and obtain none of the results of a decomposition too far advanced, which injure the [æther operation] formation of æther, but which immediately follow it. We thus obtain, at all times, a better product in greater quantity; and the termination of the production of æther will be, when the sulphuric acid, weakened and impeded by the water formed during the process, has not sufficient power to act on the alcohol.

The kind of funnel, which afforded me such assistance in producing æther by the phosphoric acid,† and which is applicable to many other chemical operations, afforded me the means of putting these opinions into practice by the following process:

A serpentine tube of glass, plunged into a vessel of cold water, was adjusted to a large tubulated retort of the same materials placed in a sand-bath. The extremity of the curved tube entered into the neck of a large receiver, from which a communication was established by means

* The proportions of sulphuric acid and spirit of wine constantly adopted, (equal parts of each) appear to be the most proper. Nevertheless, it must be remarked, that in spite of the utmost care to separate the alcohol which first passes over, the product which follows only attains the lightness and fineness of true æther towards the middle of the operation.

† Vide Annales de Chimie, No. 185. The Number preceding the one from which this is translated.

means of a siphon, with a second receiver filled with water. Some sulphuric acid concentrated to 66 degrees, six kilogrammes, for example, was introduced into the retort. A funnel with a double stop-cock was passed into the tubuline, so that the stem reached nearly to the bottom of the retort, and through the sulphuric acid. Ten kilogrammes of alcohol (at 36 degrees of the aerometer of Beaume) were rapidly introduced, which by means of the funnel passed into the acid. The mixture takes place very well, though with violence, and is less coloured in proportion as the introduction is expeditious. Distillation is commenced by a fire placed under the retort, and when about two kilogrammes are obtained, ten kilogrammes more alcohol, at 40 degrees,* are added drop by drop, regulating the quantity introduced by that which passes into the receiver. The operation is continued till fifteen kilogrammes are drawn off, and the product is limped pale, has a more agreeable æthereal odour, and without any trace of sulphuric acid or sweet oil, yielding on rectification eight kilogrammes of pure æther, and alcohol of an æthereal odour highly useful for future operations.

The liquor remaining in the retort is then of the colour of beer, and transparent; it is composed of nearly the whole quantity of acid employed, alcohol, water, and without doubt, of a certain quantity of æther ready formed.

Heated a fresh, this residuum quickly takes on a black colour; and in this state, it may, on occasion, be used in the composition of Hoffman's anodyne liquor. Or, it may be used where the alcohol can do no harm instead of the sulphuric acid, as, for example, in the formation of different salts.

---

### *To the Editors of the Medical and Physical Journal.*

Gentlemen,

THE principles of no art or science demand more deliberate discussion, than that which intimately regards the welfare of our fellow-creature. It is on this account that I have

---

* I have remarked that alcohol at 36 degrees, is the best for the ordinary preparation of sulphuric æther, and that the mixture is less coloured at that degree than when it is less dilute; whilst, for the second addition the acid being already weakened, it is preferable to employ it at 40 degrees.

